# Evaluation of long‐acting cabotegravir safety and pharmacokinetics in pregnant women in eastern and southern Africa: a secondary analysis of HPTN 084

**DOI:** 10.1002/jia2.26401

**Published:** 2025-01-02

**Authors:** Sinead Delany‐Moretlwe, Brett Hanscom, Xu Guo, Clemensia Nkabiito, Patricia Mandima, Patricia Ntege Nahirya, Juliet Mpendo, Muchaneta Bhondai‐Mhuri, Nyaradzo Mgodi, Rebecca Berhanu, Jennifer Farrior, Estelle Piwowar‐Manning, Susan L. Ford, Craig W. Hendrix, Alex R. Rinehart, James F. Rooney, Adeola Adeyeye, Raphael J. Landovitz, Myron S. Cohen, Mina C. Hosseinipour, Mark A. Marzinke, the HPTN 084 Study Team

**Affiliations:** ^1^ Wits RHI University of the Witwatersrand Johannesburg South Africa; ^2^ Statistical Centre for HIV/AIDS Research Prevention Fred Hutchinson Cancer Research Institute Seattle Washington USA; ^3^ Makerere University ‐ Johns Hopkins University Research Collaboration Kampala Uganda; ^4^ University of Zimbabwe Clinical Trials Research Centre Harare Zimbabwe; ^5^ Baylor College of Medicine Children's Foundation Uganda Kampala Uganda; ^6^ UVRI‐IAVI Entebbe Uganda; ^7^ FHI 360 Durham North Carolina USA; ^8^ Department of Pathology Johns Hopkins University School of Medicine Baltimore Maryland USA; ^9^ ViiV Healthcare Durham North Carolina USA; ^10^ Department of Medicine Johns Hopkins University School of Medicine Baltimore Maryland USA; ^11^ Gilead Sciences Foster City California USA; ^12^ Division of AIDS National Institute for Allergy and Infectious Diseases Rockville Maryland USA; ^13^ Center for Clinical AIDS Research and Education University of California Los Angeles California USA; ^14^ University of North Carolina (UNC) at Chapel Hill Chapel Hill North Carolina USA; ^15^ UNC Project‐Malawi Lilongwe Malawi

**Keywords:** cabotegravir, HIV prevention, long‐acting, PrEP, pregnancy, women

## Abstract

**Introduction:**

Long‐acting injectable cabotegravir (CAB‐LA) for pre‐exposure prophylaxis significantly reduced HIV acquisition in HPTN 084. We report on the safety and CAB‐LA pharmacokinetics in pregnant women during the blinded period of HPTN 084.

**Methods:**

Participants were randomized 1:1 to either active cabotegravir (CAB) plus tenofovir disoproxil fumarate/emtricitabine (TDF/FTC) placebo or active TDF/FTC plus CAB placebo. Pregnancy testing was performed at each visit; participants with a positive test had study product withheld and were offered open‐label TDF/FTC. Pregnancies were confirmed on two tests at least 4 weeks apart. All participants with a positive pregnancy test prior to November 5, 2020 are included in this analysis. Pregnancy incidence, maternal adverse event (AE) incidence, pregnancy outcomes (including composite outcome of spontaneous abortion <20 weeks, intrauterine foetal death or stillbirth ≥20 weeks, premature birth <37 weeks, or small for gestational age) were assessed. The apparent terminal phase half‐life (t_1/2app_) of CAB‐LA in pregnant women in HPTN 084 was compared to non‐pregnant women from the phase 2a HPTN 077 trial. Multivariable models assessed associations with t_1/2app._

**Results:**

Fifty‐seven pregnancies (30 CAB‐LA, 27 TDF/FTC) were confirmed over 3845 person‐years [py] (incidence 1.5/100 py, 95% CI 1.1−1.9). CAB‐LA group participants had a median 342 days (IQR 192, 497) of CAB‐LA exposure prior to pregnancy detection. Grade 2 or higher maternal AE incidence did not differ by study arm (CAB 157, 95% CI 91−271 per 100 py vs. TDF/FTC 217, 95% CI 124–380 per 100 py; *p* = 0.256). Most pregnancies (81%) resulted in live births (25 CAB‐LA, 22 TDF/FTC). Composite poor pregnancy outcomes did not differ significantly by group (CAB 6/30 vs. TDF/FTC 4/27; *p* = 0.476). No congenital anomalies were observed. The CAB t_1/2app_ geometric mean was 52.8 days (95% CI 40.7−68.4) in pregnant women compared to 60.3 days (95% CI 47.7−76.3; *p* = 0.66) in non‐pregnant women; neither pregnancy nor body mass index were significantly associated with t_1/2app_.

**Conclusions:**

CAB‐LA concentrations post‐cessation of injections were generally well tolerated in pregnant women. The t_1/2app_ was comparable between pregnant and non‐pregnant women. Ongoing studies will examine the safety and pharmacology of CAB‐LA in women who choose to continue CAB‐LA through pregnancy and lactation.

## 1 INTRODUCTION

Reproductive‐age women in sub‐Saharan Africa (SSA) are disproportionately affected by HIV, accounting for 52% of new HIV acquisitions despite representing 24% of the population [1]. Preventing HIV in susceptible populations who are also at risk for pregnancy is a priority for reducing perinatal HIV transmission, and maternal and infant morbidity and mortality. The World Health Organization (WHO) recommends pre‐exposure prophylaxis (PrEP) for HIV prevention, and efforts have been made to scale up oral PrEP in the region. In SSA where fertility rates are two‐fold higher than any other region in the world, women using PrEP may experience pregnancy [2]. As PrEP access expands, data on the safety and optimal dosing of PrEP agents in pregnancy and lactation are essential.

Long‐acting injectable cabotegravir (CAB‐LA) is a novel integrase strand transfer inhibitor (INSTI) that was shown in HPTN 084 to substantially reduce HIV acquisition in individuals who were assigned female at birth when administered every 8 weeks [3]. No evidence of the effects of CAB‐LA on embryo‐foetal development has been observed in pre‐clinical studies. In rat pre‐ and post‐natal development studies, delays in parturition were observed at extremely high cabotegravir (CAB) concentrations (1000 mg/kg/day), well in excess of doses used in humans, but the significance of this in humans is uncertain [4]. Women enrolling in the HPTN 084 trial, however, were required to avoid pregnancy and to use a reliable modern contraceptive method. No significant drug interactions between CAB‐LA and hormonal contraceptives were expected [5, 6]. Only long‐acting reversible contraceptives (LARCs) with a failure rate of less than 1% were allowed following initial reports of neural tube defects in the context of peri‐conception dolutegravir use in women living with HIV (WLH) [7]. Despite these requirements, pregnancies occurred during the trial [3]. Here, we report on updated safety, clinical outcomes and CAB‐LA pharmacokinetics (PK) in women who became pregnant during the blinded phase of HPTN 084.

## 2 METHODS

### 2.1 Study design and participants

The study has been described in detail elsewhere [3]. Briefly, 3224 participants from Botswana, Eswatini, Kenya, Malawi, South Africa, Uganda and Zimbabwe were enrolled between November 2017 and November 2020 into the blinded portion of the trial. Consenting participants were enrolled if they were HIV negative, between the ages of 18–45 years, female sex at birth, sexually active, not pregnant or breastfeeding, in good health and with no contra‐indications to either CAB‐LA or oral tenofovir disoproxil fumarate/emtricitabine (TDF/FTC). All participants of reproductive potential were required to use an LARC, including contraceptive injections, implants or intra‐uterine devices, following a protocol amendment in May 2018. Site staff were trained in contraceptive counselling and on‐site contraceptive method provision. Participants who sought contraception from local health facilities were required to provide proof of LARC use. Participants who declined LARC use during the trial had study product temporarily withheld and were offered open‐label TDF/FTC.

### 2.2 Procedures

Participants completed a 5‐week oral lead‐in (Step 1) with blinded oral pills to establish tolerability prior to starting the blinded injection phase of the study (Step 2). All participants then received the first two blinded injections 4 weeks apart, and then every 8 weeks thereafter along with blinded oral pills to be taken daily. Participants were tested for pregnancy and HIV prior to product dispensation. Injectable study product was withheld if a participant had a positive urine pregnancy test, and open‐label TDF/FTC was initiated while the pregnancy was confirmed on a second test 4 weeks later. Confirmatory pregnancy tests were required to avoid the potential for overdiagnosis of chemical pregnancies due to frequent testing [8].

If the pregnancy was confirmed, the participant was unblinded per United States Food and Drug Administration requirements, open‐label TDF/FTC was continued throughout pregnancy and breastfeeding, and quarterly follow‐up visits were continued through to pregnancy outcome. Participants continued to receive a comprehensive sexually transmitted infection (STI), including HIV, prevention package. All pregnant participants were counselled regarding the risks and benefits of CAB‐LA exposure during pregnancy and had or were referred for a first‐trimester ultrasound for foetal anomaly assessment and further antenatal care in accordance with local guidelines. Ultrasound assessments captured on case report forms were used to estimate gestational age (GA). Adverse events (AEs) were assessed at each visit using the Division of AIDS Table for Grading the Severity of Adult and Paediatric Adverse Events (version 2.1, July 2017) [9]. Weight was collected at protocol‐specified visits using a standardized procedure. Blood samples were collected at each visit for HIV testing, safety assessments and plasma storage. Testing for *T. vaginalis* (Osom Trichomonas Rapid Test ®, Sekisui Diagnostics, MA, USA or wet preparation), *C. trachomatis* and *N. gonorrhoea* using nucleic acid amplification testing, and syphilis serology was performed every 6 months. STIs were treated per local guidelines. All pregnancy outcomes, including congenital anomalies, were ascertained by participant report and medical record review when available and confirmed by study staff assessment. Infant birth and growth parameters were confirmed using medical records from the time of delivery. Following pregnancy outcome and/or cessation of breastfeeding, the participant could return unblinded to her originally randomized study product. Plasma CAB‐LA concentrations were quantified at the HIV Prevention Trials Network Laboratory Center (Baltimore, MD) using liquid chromatography–tandem mass spectrometry, with a lower limit of quantification (LLOQ) of 25.0 ng/ml [10].

### 2.3 Statistical outcomes and analysis

Participant data for all participants with a positive pregnancy test prior to November 5, 2020 are included in this analysis. Person‐years were calculated as the time from enrolment up to the first positive pregnancy test or the last visit prior to the end of the blinded period. Pregnancy incidence was estimated for all participants with a positive pregnancy test as well as for pregnancies confirmed on two pregnancy tests approximately 4 weeks apart, with 95% confidence intervals calculated using the exact Poisson method. Baseline characteristics of participants with at least one positive pregnancy test were compared to those with no pregnancy using *t*‐tests for continuous variables (VOICE risk score and number of vaginal sex episodes) and chi‐square tests for categorical variables [11].

Pregnancy outcomes were evaluated in all participants who became pregnant and summarized using descriptive statistics, stratified by pregnancy confirmation status.  Outcomes were categorized as follows:  live birth (≥37 weeks); premature birth (<37 weeks); spontaneous abortion (<20 weeks); stillbirth or intrauterine foetal demise (≥20 weeks); therapeutic or elective abortion.  A composite pregnancy safety outcome measure is defined as the occurrence of any of the following: spontaneous abortion (<20 weeks of gestation), stillbirth (≥20 weeks of gestation), preterm delivery (<37 weeks of gestation in live‐born babies) or small for gestational age (SGA; weight <10th percentile for GA, adjusted for sex), was calculated and compared by study arm for confirmed pregnancies [12]. GA was estimated using the earliest ultrasound exam when available, or the last menstrual period date when ultrasound was not available [13]. GA was centrally re‐estimated based on foetal biometry and using the Intergrowth‐21 tables. Infant growth parameters at birth were standardized using the WHO z‐score adjusted for sex at birth [14].

Incidence rates for grade 2 or higher clinical and laboratory AE during pregnancy were estimated for participants with confirmed pregnancies who received at least one injection prior to pregnancy. Weight gain was calculated using weight at the visit where the pregnancy was first detected through to the most recent visit preceding the pregnancy outcome date. A linear mixed model with pregnancy time, study arm and time by arm interaction was fitted to evaluate mean weight gain per randomized study product.

CAB‐LA PK assessment was restricted to participants randomized to the CAB‐LA arm. The apparent terminal half‐life (t_1/2app_) was estimated in participants who received at least one injection and had at least three CAB‐LA measurements above the LLOQ after pregnancy confirmation. Individual log‐linear regression curves were fitted for plasma CAB‐LA concentrations from the visit following the last injection prior to the first positive pregnancy date to the last available sample up to 6 months after the date of study unblinding (May 05, 2021), or 24 weeks after pregnancy end‐date, whichever came first; this defined the PK period for this analysis. CAB‐LA concentration decline over time was estimated using terminal slope (K_a_) derived from natural log‐linear regression and t_1/2app_ was estimated as the natural log transformation of −ln(2)/K_a_. A sensitivity analysis was performed and participants with t_1/2app_ estimates with adjusted *R* ^2^ of ≥ 0.85 were included in subsequent regression analyses. The t_1/2app_ in HPTN 084 participants was compared to t_1/2app_ in non‐pregnant women from the phase 2a HPTN 077 clinical trial (Cohort 2, 600 mg dose administered on the same dosing schedule as used in HPTN 084), where comparable sample selection criteria (at least three samples after cessation of injections with CAB‐LA concentrations above the LLOQ) were used to determine terminal decay [10]. The association between t_1/2app_ and participant baseline characteristics, including body mass index (BMI), age, weight, race and pregnancy status, was assessed in univariate and multivariate log‐linear regression models. BMI was dichotomized (27.2 kg/m^2^) according to median concentrations observed in the phase 2a HPTN 077 study. All variables except for weight were included in the multivariate model; weight was excluded to avoid collinearity with BMI. All analyses were performed using SAS 9.4 (Cary, NC).

## 3 RESULTS

### 3.1 Participant characteristics

Of 3224 participants enrolled in HPTN 084, 74 participants had at least one positive pregnancy test during follow‐up (38 CAB‐LA, 36 TDF/FTC) (Table 1). Median age was 24 (interquartile range [IQR] 21, 27) years at enrolment, with most (84%) reporting previous pregnancies, and a median of one (IQR 1.0, 2.0) previous live birth. Participants with a positive pregnancy test were mostly single (38%) or not living with their primary partner (31%), had secondary‐level education (72%), were unemployed (64%), came from Zimbabwe (36%) or Uganda (35%) and reported injectable contraceptive use (53%) (Table 1). They were more likely than non‐pregnant participants to be younger, living with a partner, have engaged in transactional sex, use contraceptive pills or intrauterine contraceptive device (IUCD) for contraception, and have had a previous pregnancy and live birth (Table 1).

**Table 1:** Baseline characteristics, by positive pregnancy test status

	At least one pregnancy test positive *N* = 74 *n* (%)	No pregnancy test positive *N* = 3150 *n* (%)	*p*‐value^a^
Study arm			0.822
TDF/FTC	36/74 (49%)	1574/3150 (50%)	
Cabotegravir	38/74 (51%)	1576/3150 (50%)	
Country			<0.001
Botswana	3/74 (4%)	88/3150 (3%)	
Kenya	4/74 (5%)	62/3150 (2%)	
Malawi	6/74 (8%)	218/3150 (7%)	
South Africa	8/74 (11%)	1300/3150 (41%)	
Eswatini	0/74 (0%)	160/3150 (5%)	
Uganda	26/74 (35%)	569/3150 (18%)	
Zimbabwe	27/74 (36%)	752/3150 (24%)	
Median age (years) (IQR)	24.0 (21, 27)	25.0 (22, 30)	0.006
Age≤25	45/74 (61%)	1804/3150 (57%)	0.543
Marital status			<0.001
Married/civil union/legal partnership	8/74 (11%)	335/3150 (11%)	
Living with primary partner	14/74 (19%)	210/3150 (7%)	
Not living with primary partner	23/74 (31%)	1705/3150 (54%)	
Single/divorced/widowed	28/74 (38%)	891/3150 (28%)	
Other	1/74 (1%)	8/3150 (<1%)	
Highest education			0.075
No schooling	1/74 (1%)	31/3150 (1%)	
Primary school	18/74 (24%)	488/3150 (15%)	
Secondary school	53/74 (72%)	2283/3150 (72%)	
Technical training	1/74 (1%)	88/3150 (3%)	
College/university or higher	1/74 (1%)	260/3150 (8%)	
Employment status			0.193
Not employed	47/74 (64%)	2299/3150 (73%)	
Part‐time employment	13/74 (18%)	408/3150 (13%)	
Full‐time employment	14/74 (19%)	442/3150 (14%)	
In the prior month^b^			
Partner HIV positive or unknown	56/74 (66%)	2235/3136 (71%)	0.309
≥2 Sex partners	48/73 (66%)	1707/3136 (54%)	0.149
Transactional sex	47/73 (64%)	1266/3136 (40%)	<0.001
Anal sex	6/73 (8%)	179/3136 (6%)	0.310
Median VOICE risk score at screening (IQR)	6.0 (5, 7)	6.0 (5, 7)	0.597
Body mass index ≥30 kg/m^2^	21/74 (28%)	874/3150 (28%)	0.904
Contraceptive method at baseline			<0.001
Oral contraceptive pills^c^	4/74 (5%)	33/3149 (1%)	
Intrauterine device (IUCD)	8/74 (11%)	134/3149 (4%)	
Injectable—DMPA/NET‐EN	39/74 (53%)	1970/3149 (63%)	
Implants	22/74 (30%)	989/3149 (31%)	
Sterilization	1/74 (1%)	23/3149 (1%)	
Previous pregnancy	62/74 (84%)	30/3150 (1%)	<0.001
Median number of previous live births (IQR)	1.0 (1.0, 2.0)	0.0 (0.0, 0.0)	<0.001
Sexually transmitted infections			
*C. trachomatis*	19/74 (26%)	585/3115 (19%)	0.135
*N. gonorrhoeae*	5/74 (7%)	205/3115 (7%)	0.952
*Trichomonas vaginalis*	4/73 (5%)	266/3060 (9%)	0.334
Syphilis	3/74 (4%)	100/3145 (3%)	0.673

Abbreviations: DMPA/NET‐EN, depot medroxyprogesterone acetate/norethisterone enanthate; IQR, interquartile range; TDF/FTC, tenofovir disoproxil fumarate/emtricitabine.

^a^ Chi‐square *p*‐values for categorical variables, or *t*‐test *p*‐values for continuous variables were shown here.

^b^ Responses are available for 3209/3211; responses are missing for 15 participants (CAB 5, TDF/FTC 10).

^c^ Oral contraceptive pills were not allowed as a method of contraception following safety concerns regarding dolutegravir and neural tube defects; the protocol was modified to only allow LARC use.

### 3.2 Pregnancy incidence

Overall, 74 women had at least one positive pregnancy test for a total of 78 pregnancies reported during 3845‐person years of follow‐up (incidence 2.0/100 py, 95% CI 1.6−2.5) (Table 2). Twenty‐one pregnancies were not confirmed at a second visit and had outcomes categorized as ectopic pregnancy (1 CAB‐LA, 1 TDF/FTC), spontaneous abortion (3 CAB‐LA, 5 TDF/FTC) or therapeutic abortion (5 CAB‐LA, 5 TDF/FTC); there was no outcome for one participant (1 TDF/FTC).

**Table 2:** Cumulative pregnancy incidence and outcomes, by study group

	CAB‐LA *N* = 1614 *n* (%)	TDF/FTC *N* = 1610 *n* (%)	Overall *N* = 3224
**Pregnancy incidence**			
Total number of pregnancies	39	39	78
Person‐years	1919.9	1924.9	3844.8
Incidence rate	2	2	2
95% CI for the incidence rate	1.4, 2.8	1.4, 2.8	1.6, 2.5
Number of confirmed pregnancies^a^	30	27	57
Person‐years	1919.9	1924.9	3844.8
Incidence rate of confirmed pregnancy	1.6	1.4	1.5
95% CI for the incidence rate	1.1, 2.2	0.9, 2.0	1.1, 1.9
**Pregnancy outcomes**	40	39	79
Outcomes for pregnancies that ended prior to confirmation	9	12	21
No obtainable outcome	0/9 (0%)	1/12 (8%)	1/21 (5%)
Ectopic pregnancy	1/9 (11%)	1/12 (8%)	2/21 (10%)
Spontaneous abortion (<20 weeks)	3/9 (33%)	5/12 (42%)	8/21 (38%)
Therapeutic/elective abortion	5/9 (56%)	5/12 (42%)	10/21 (48%)
Outcomes for confirmed pregnancies^b^	31	27	58
No obtainable outcome	1/31 (3%)	0/27 (0%)	1/58 (2%)
Ectopic pregnancy	0/31 (0%)	1/27 (4%)	1/58 (2%)
Spontaneous abortion (<20 weeks)	2/31 (6%)	2/27 (7%)	4/58 (7%)
Therapeutic/elective abortion	2/31 (6%)	1/27 (4%)	3/58 (5%)
Stillbirth/intrauterine foetal demise (≥ 20 weeks)	1/31 (3%)	1/27 (4%)	2/58 (3%)
Preterm delivery (<37 weeks)^b^	3/31 (10%)	0/27 (0%)	3/58 (5%)
Full‐term delivery (≥37 weeks)	22/31 (71%)	22/27 (81%)	44/58 (76%)

Abbreviations: CAB‐LA, cabotegravir; CI, confidence interval; TDF/FTC, tenofovir disoproxil fumarate/emtricitabine.

^a^ Confirmed pregnancies defined by two positive pregnancy tests at least 4 weeks apart.

^b^ Fifty‐seven confirmed pregnancies occurred in 57 unique participants and resulted in 58 distinct pregnancy outcomes (there was one set of twins).

The remaining 57 pregnancies (30 CAB‐LA, 27 TDF/FTC) were confirmed on a second test at a subsequent visit and form the basis for the remainder of this analysis (Table 2). Confirmed pregnancy incidence was 1.5/100 py (95% CI 1.1−1.9) and did not vary significantly by study arm. Median GA at pregnancy detection was 6.06 weeks (IQR: 3.4, 8.7) (CAB‐LA 6.9, IQR: 3.7, 9.0; TDF/FTC 5.4, IQR 3.4, 7.1). Seven confirmed pregnancies were detected in Step 1 (3 CAB‐LA, 4 TDF/FTC) and 50 in Step 2 (27 CAB‐LA, 23 TDF/FTC). Participants who became pregnant in Step 2 received a median of 6 injections (IQR: 3, 8) prior to the first positive pregnancy test; the median number of blinded injections was comparable between study groups (CAB‐LA median 5, IQR: 3, 9; TDF/FTC median 6, IQR: 3, 8). Participants randomized to CAB‐LA had a median of 342 days (IQR: 192, 497) exposure prior to the first positive pregnancy result.

### 3.3 Pregnancy and infant outcomes

Of the 57 confirmed pregnancies, 58 pregnancy outcomes were observed, including one twin pregnancy and one unobtainable outcome (Table 2). Pregnancy outcomes did not differ substantially by study arm. Of the pregnancy outcomes, 5% (3/58) were reported as elective terminations, 9% (5/58) were early pregnancy losses and 3% (2/58) were reported as intra‐uterine foetal demise or stillbirth. In the latter two cases, one occurred in a participant with pre‐eclampsia who delivered in a hospital setting, and one occurred in a participant who delivered at home and was assessed as a late miscarriage (22 weeks GA) by the investigator.

**Figure 1:**
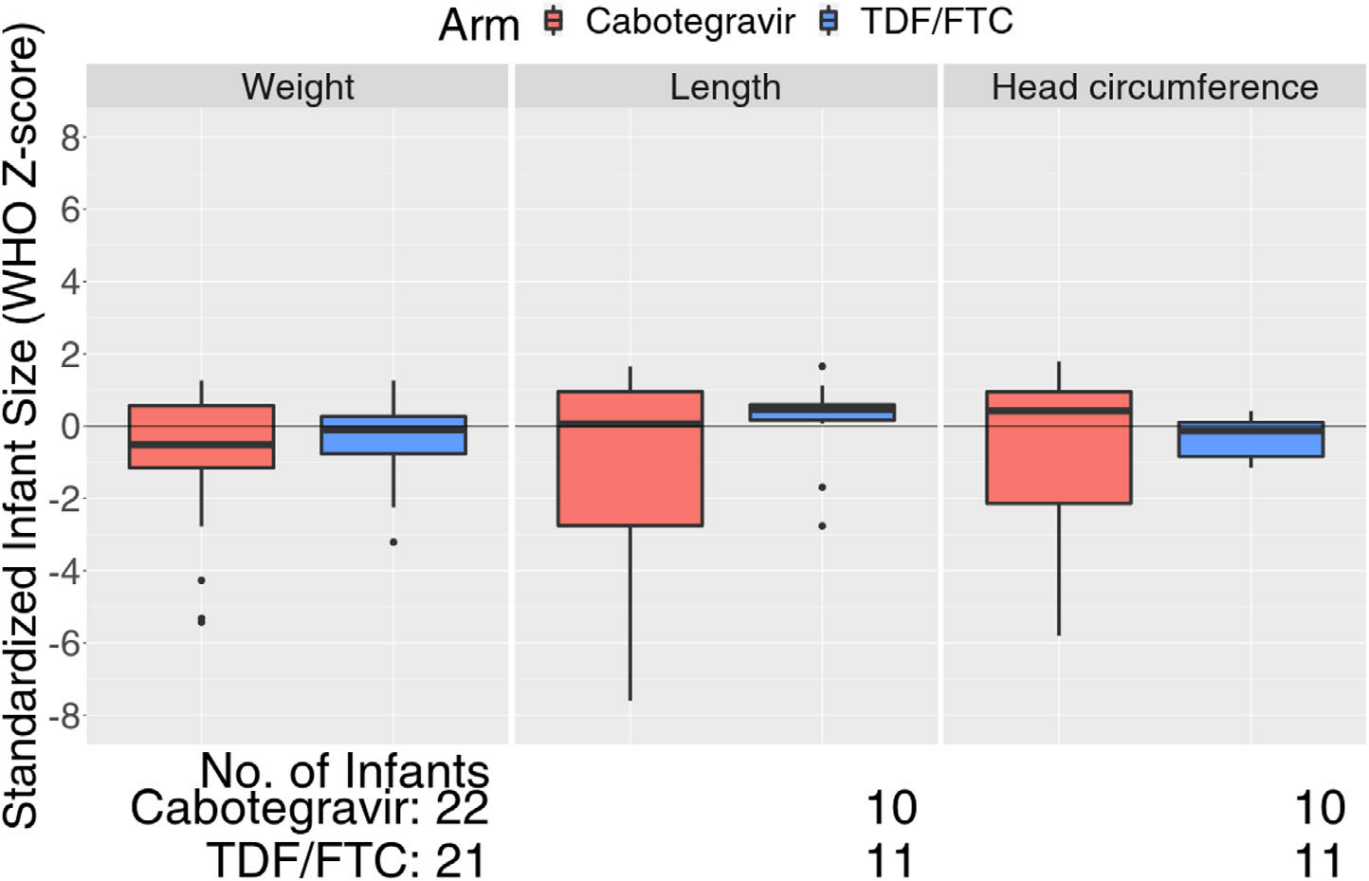
Box plots of standardized infant growth measures at birth, by study arm.

Most pregnancies resulted in live births (25 CAB‐LA, 22 TDF/FTC) (Table 2). Of the live births, three were defined as pre‐term delivery (all CAB‐LA) and included twin births, delivered in hospital by normal vaginal delivery at 31 weeks GA following premature rupture of membranes with no further delivery complications. Both were SGA. The other pre‐term delivery (28 weeks GA) was hospital‐based, following premature rupture of membranes and foetal distress. A live infant was delivered but demised within the first 24 hours and was classified as a neonatal death. The investigator did not consider this death product‐related. Among the remaining full‐term live births (22 CAB‐LA, 22 TDF/FTC), 93% were delivered by a medical attendant at a hospital, clinic or health centre (20 CAB‐LA, 21 TDF/FTC), while the remainder (2/44) were delivered at home (1 CAB‐LA, 1 TDF/FTC) or by a traditional birth attendant (1 TDF/FTC). Of these full‐term live births, 6/44 (14%) were delivered by caesarean section (5 CAB‐LA, 1 TDF/FTC). Indications for caesarean section were foetal distress (*n* = 2), prolonged labour (*n* = 2), cephalopelvic disproportion (*n* = 1) (CAB‐LA) and oligohydramnios (*n* = 1) (TDF/FTC). Among the live births, 3/47 (11%) were considered SGA (3 CAB‐LA). There were no differences in infant growth parameters at delivery by study arm (Figure 1). No congenital anomalies were reported among births that could be assessed.

Using the derived composite adverse pregnancy outcome for all confirmed pregnancies (*n* = 57), 6/30 (20%) confirmed pregnancies in the CAB‐LA group were associated with an adverse outcome compared with the 3/27 (11.1%) pregnancies in the TDF/FTC group; the difference was not statistically different (*p* = 0.476).

### 3.4 AEs during pregnancy

The incidence of grade 2 or higher AEs in participants who received at least one injection was 157/100 py (95% CI 91–271/100 py) in the CAB‐LA arm compared to 217/100 py (95% CI 124−380/100 py) in the TDF/FTC arm (*p* = 0.256) (Table 3). The most frequent AE reported was urinary tract infection (29.8/100 py CAB‐LA; 29.9/100 py TDF/FTC). There was no difference in weight gain during pregnancy by study group (CAB‐LA mean 0.94 kg/month, 95% CI 0.53−1.4 vs. TDF/FTC mean 0.75 kg/month, 95% CI 0.28−1.2, *p*‐value = 0.48).

**Table 3:** Adverse events during pregnancy, by randomized study group

Adverse event	CAB‐LA: Events	CAB‐LA incidence (per 100 py)	CAB‐LA 95% CI	TDF/FTC: Events	TDF/FTC incidence (per 100 py)	TDF/FTC 95% CI	*p*‐value
Any grade 2 + AE	21	156.5	(90.6, 270.6)	29	216.7	(123.7, 379.6)	0.256
Urinary tract infection	4	29.8	(11.7, 75.8)	4	29.9	(12.0, 74.4)	0.997
Chlamydia infection	4	29.8	(9.6, 93.0)	3	22.4	(7.7, 65.0)	0.709
Premature rupture of membranes	2	14.9	(3.7, 59.6)	0	0.0	NA	0.489
Decreased creatinine clearance	0	0.0	−	3	22.4	(7.2, 69.5)	0.115
Myalgia	0	0.0	−	3	22.4	(7.2, 69.5)	0.115
Increased blood glucose	0	0.0	−	2	14.9	(3.7, 59.8)	0.243
Genital candidiasis	0	0.0	−	2	14.9	(3.7, 59.8)	0.243
Dysfunctional uterine bleeding	1	7.5	(1.1, 52.9)	0	0.0	−	1.000
Foetal distress syndrome	1	7.5	(1.1, 52.9)	0	0.0	−	1.000
Hyperemesis gravidarum	1	7.5	(1.1, 52.9)	0	0.0	−	1.000
Hypophosphataemia	1	7.5	(1.1, 52.9)	0	0.0	−	1.000
Pregnancy‐induced hypertension	1	7.5	(1.1, 52.9)	0	0.0	−	1.000
Pre‐eclampsia	1	7.5	(1.1, 52.9)	0	0.0	−	1.000
Starvation	1	7.5	(1.1, 52.9)	0	0.0	−	1.000
Syphilis	1	7.5	(1.1, 52.9)	0	0.0	−	1.000
Upper respiratory infection	1	7.5	(1.1, 52.9)	0	0.0	−	1.000
Increased blood amylase	1	7.5	(1.1, 50.0)	1	7.5	(1.1, 49.4)	0.999
Oligohydramnios	1	7.5	(1.1, 51.2)	1	7.5	(1.1, 50.3)	0.999
Decreased blood glucose	0	0.0	−	1	7.5	(1.1, 53.1)	0.499
Constipation	0	0.0	−	1	7.5	(1.1, 53.1)	0.499
Dermatitis	0	0.0	−	1	7.5	(1.1, 53.1)	0.499
Glycosuria	0	0.0	−	1	7.5	(1.1, 53.1)	0.499
Nasopharyngitis	0	0.0	−	1	7.5	(1.1, 53.1)	0.499
Postoperative wound infection	0	0.0	−	1	7.5	(1.1, 53.1)	0.499
Postpartum haemorrhage	0	0.0	−	1	7.5	(1.1, 53.1)	0.499
Prolonged pregnancy	0	0.0	−	1	7.5	(1.1, 53.1)	0.499
Thermal burn	0	0.0	−	1	7.5	(1.1, 53.1)	0.499
Vaginal discharge	0	0.0	−	1	7.5	(1.1, 53.1)	0.499

Abbreviations: CAB‐LA, cabotegravir; CI, confidence interval; py, person‐year; TDF/FTC, tenofovir disoproxil fumarate/emtricitabine.

Five CAB‐LA participants and one TDF/FTC participant reported a combined total of 10 grade 2 or higher AEs (7 CAB‐LA, 3 TDF) associated with pregnancy, puerperium and perinatal conditions. All but one of these participants delivered live infants; the remaining participant (CAB‐LA) was diagnosed with pre‐eclampsia which resulted in a stillbirth noted above. Only one of these AEs (grade 4) in a participant (CAB‐LA) who had an emergency caesarean section for foetal distress was considered by the investigator as study product‐related. The participant delivered a full‐term live infant who recovered well. No additional risk factors were reported.

There were no maternal deaths during pregnancy or within 42 days after pregnancy, and no HIV acquisitions were detected during pregnancy.

### 3.5 CAB‐LA concentrations during pregnancy

**Figure 2:**
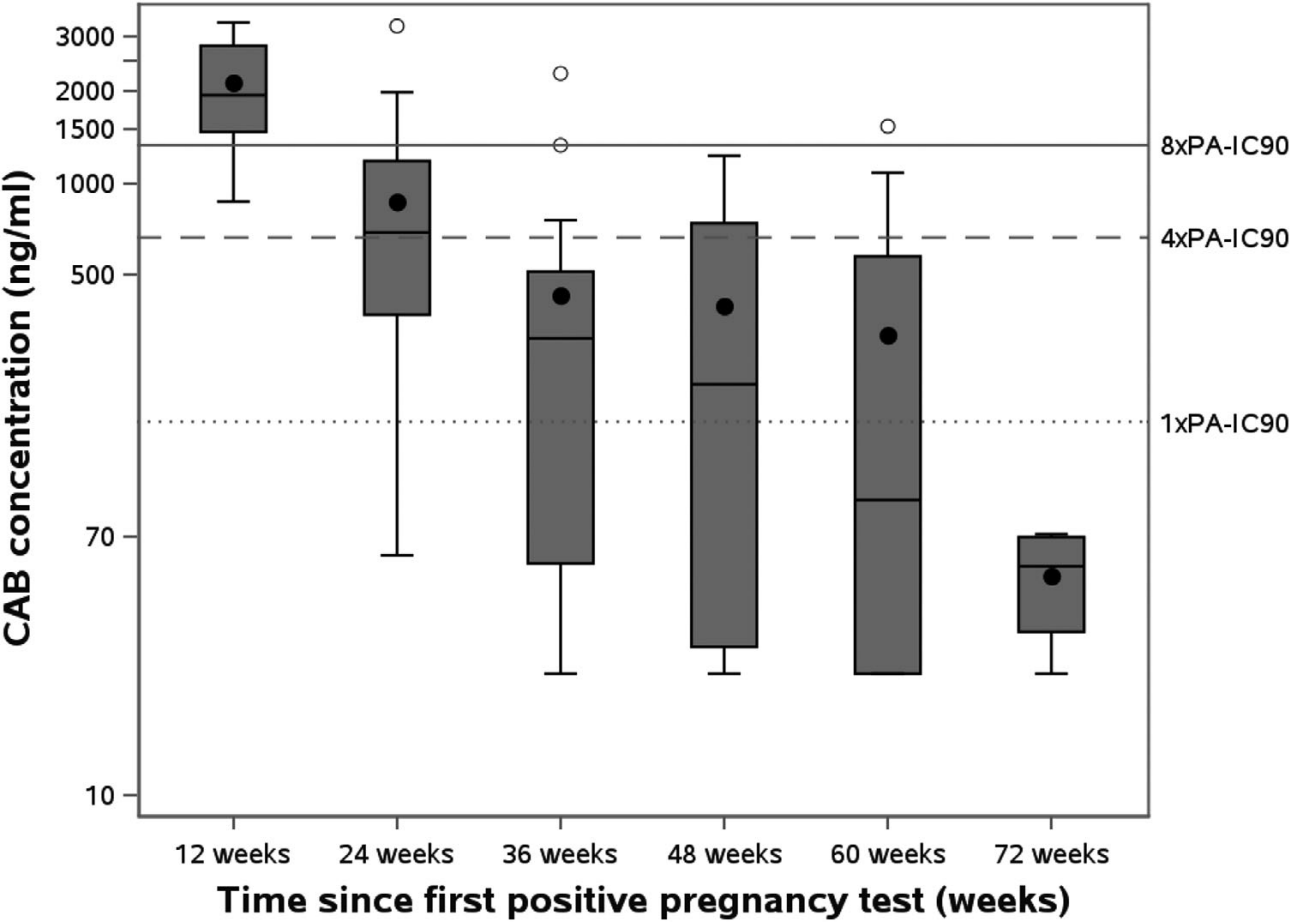
Geometric mean CAB‐LA concentrations following first positive pregnancy date in study participants who received at least one injection of CAB‐LA (*n* = 27). The in vitro protein‐adjusted 90% CAB‐LA inhibitory concentration (PA‐IC90) is 166 ng/ml [28]. The assay LLOQ is 25.0 ng/ml.

**Figure 3:**
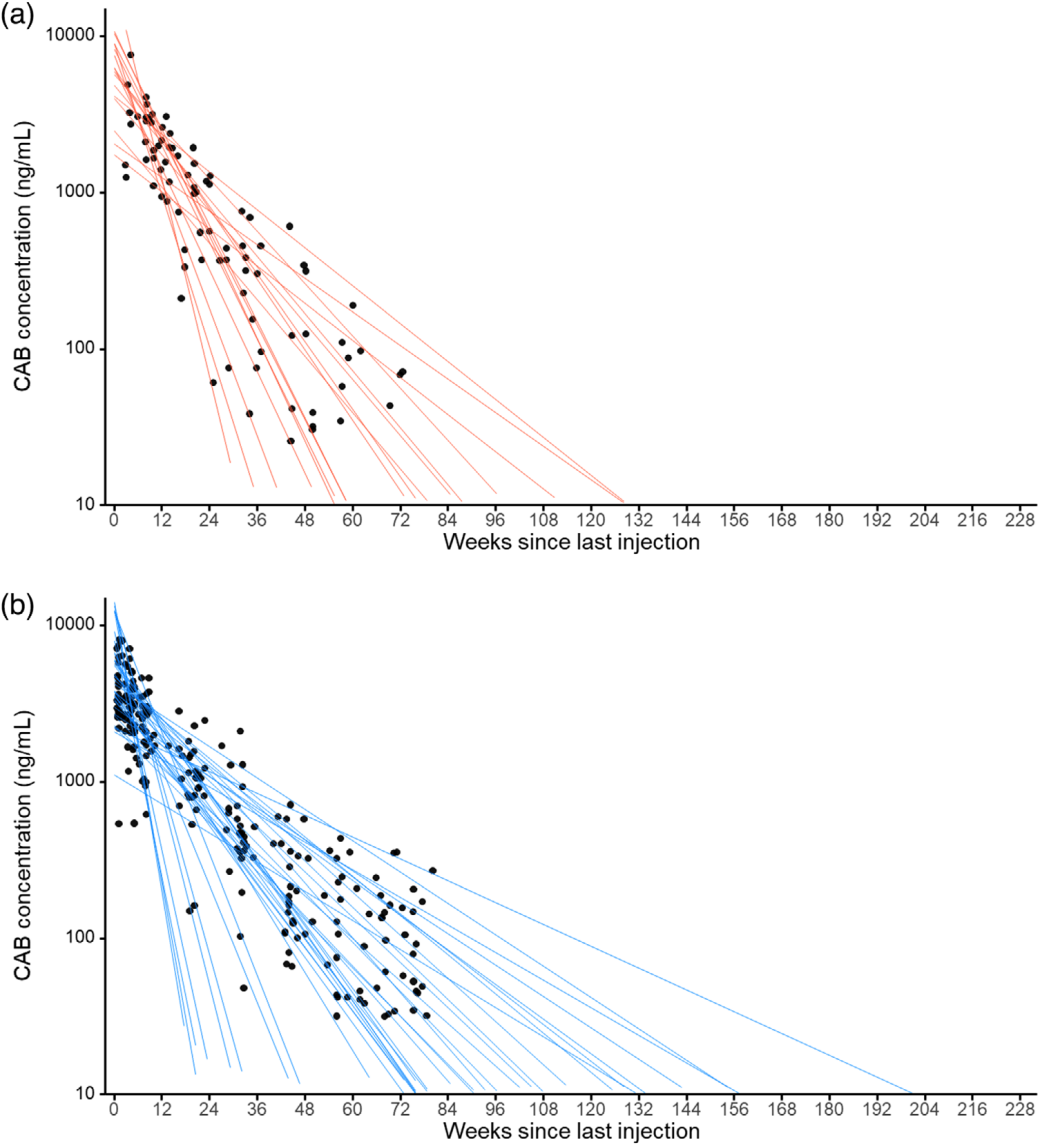
Individual participant log‐linear regression curves of plasma CAB‐LA concentrations from the visit after last injection to the last available quantifiable sample in (a) pregnant women from HPTN 084 (*n* = 17) and (b) non‐pregnant women from HPTN 077 (*n* = 35). Curves were fitted for each individual extrapolated to the intersection with LLOQ and all extended beyond the observed concentrations.

Of the 27 participants randomized to the CAB‐LA group who received at least one injection prior to pregnancy, geometric mean CAB‐LA concentrations through 72 weeks after the first positive pregnancy date are presented (Figure 2). Participants with t_1/2app_ estimates with adjusted *R* ^2^ of ≥ 0.85 were included in subsequent analyses, which included 17 women who had at least three CAB‐LA measurements post‐pregnancy confirmation from HPTN 084 and 35 non‐pregnant women from HPTN 077 who received at least one injection of 600 mg CAB‐LA. Differences in terminal decay were not statistically significant when comparing cohorts; the geometric mean t_1/2app_ was 52.8 days (95% CI 40.7−68.4) in pregnant women and 60.4 days (95% CI 47.7−76.3; *p* = 0.66) in non‐pregnant women (Figure 3).

In univariate and multivariate regression analysis, no parameter was significantly associated with t_1/2app_ (Table 4). In the multivariate regression analysis, geometric fold change in CAB‐LA t_1/2app_ was 0.80 in women with confirmed pregnancy compared to non‐pregnant women (*p* = 0.354). The geometric mean fold change in CAB t_1/2app_ was 1.35 for women with BMI >27.2 compared to women with BMI ≤ 27.2 (*p* = 0.157). A comparison of a multivariate regression model (all variables) to a multivariate model without the inclusion of BMI and pregnancy status showed that 4.2% of the variability in CAB‐LA t_1/2app_ was attributed to BMI.

**Table 4:** Association of baseline characteristics with CAB‐LA t_1/2app_

	Univariate linear regression		Multivariate linear regression	
Parameter	Coefficient (95% CI)	*p*‐value	Coefficient (95% CI)	*p*‐value
BMI (>27.2 vs. ≤27.2)	1.27 (0.89, 1.81)	0.176	1.35 (0.89, 2.07)	0.157
Age (years)	1.01 (0.99, 1.03)	0.550	0.99 (0.97, 1.02)	0.679
Weight (kg)	1.01 (1.00, 1.02)	0.052	NA	NA
Pregnancy status (pregnant vs. non‐pregnant)	0.87 (0.60, 1.27)	0.476	0.80 (0.50, 1.29)	0.354
Number of injections	0.99 (0.91, 1.07)	0.773	1.01 (0.93, 1.11)	0.759

Abbreviations: BMI, body mass index; t_1/2app_, apparent terminal phase half‐life.

## 4 DISCUSSION

We saw no statistically significant differences in pregnancy incidence and outcomes, maternal AEs or infant growth parameters by originally randomized group in women who became pregnant during the blinded portion of HPTN 084. A composite outcome aimed at capturing negative outcomes overall when specific adverse outcomes are infrequent also did not vary significantly by study group. Across both study arms, no HIV acquisition occurred during the pregnancy period. Although the data set is small, the frequencies of spontaneous abortion, stillbirth and pre‐term delivery are comparable with or less than observed rates in studies of oral and vaginal PrEP in pregnant women in eastern and southern Africa where comparisons were made with placebo or unexposed populations [15, 16, 17]. For example, 21–32% of pregnancies in the placebo group of previous PrEP studies ended in spontaneous abortion. The spontaneous abortion data should be interpreted cautiously for two reasons. First, participants were tested for pregnancy frequently. Studies using urine βHCG tests have shown that 22% of pregnancies may end before clinical detection. When including clinically recognized spontaneous abortions, rates may be as high as 30% in the absence of any medications [8]. Second, termination of pregnancy is associated with legal restrictions and social taboos in most of the HPTN 084 countries. Some elective terminations may have been misclassified as spontaneous abortion because participants chose not to disclose.

Most pregnancies ended in a live full‐term birth. Although CAB‐LA injections were held after the first positive pregnancy test, pregnancies were exposed to CAB‐LA during the developmentally sensitive peri‐conception period, and subsequently to residual CAB‐LA concentrations throughout the pregnant and postpartum periods. CAB‐LA has been shown in rats to cross the placenta, and data from the recent case study of CAB‐rilpivirine injections in a pregnant woman with HIV showed a cord‐maternal blood ratio of 1.27 [4, 18]. Despite CAB‐LA exposure *in utero*, there was no evidence of a substantial impact on foetal and infant growth parameters at birth. Eleven percent of the live births were assessed as SGA, and this is consistent with background SGA prevalence rates in low‐ and middle‐income country settings [19]. SGA may reflect constitutionally small infants, growth restriction and/or pre‐term birth. Although median growth parameters were similar across study groups, CAB‐LA‐exposed infants had more variation in head circumference and length. Infant growth is influenced by a range of maternal, pregnancy and infant factors including maternal height, weight gain, BMI, hypertension, history of previous low birth weight infant and foetal sex [20]. Studies in pregnant women suggest that women prescribed antiretroviral therapy (ART) are more likely than women with HIV not on ART to deliver SGA infants, but the evidence is mixed regarding the effects of HIV and/or ART on birth size [21]. To date, studies have not shown an increased risk of SGA associated with INSTI‐based regimens [22]. More data are needed to determine whether CAB‐LA has any excess influence on infant growth.

Residual CAB‐LA concentrations appeared to be well‐tolerated by women during pregnancy. The incidence of grade 2 or higher AE was greater in the TDF/FTC group, although this was not statistically significant and did not appear to be driven by excess events in any one organ class. AEs associated with pregnancy, for example, hypertension or eclampsia, were isolated events. There are concerns about the cardiometabolic effects of INSTIs on pregnant WLH [23]. Some observational studies have shown an increased risk for hypertension in WLH on INSTI compared to non‐INSTI regimens [24]. Women with hypertensive disorders in pregnancy are more likely to have negative pregnancy outcomes, including pre‐term birth and neonatal mortality. Excess gestational weight gain may also contribute to hypertension in pregnancy. INSTI‐based regimens have been associated with weight gain in both pregnant and non‐pregnant WLH [25, 26]. While CAB‐LA was observed to be associated with an initial weight gain of <1 kg in women HPTN 084, significant treatment group differences in weight gain did not persist during follow‐up [3]. Similarly, in our analysis of gestational weight gain, both groups of participants gained < 1 kg on average per month of pregnancy, and no significant differences in weight gain were observed. Ultimately, these metabolic effects require investigation in larger cohorts and in women without HIV who receive CAB‐LA injections throughout pregnancy.

No differences in CAB t_1/2app_ were observed in pregnant compared to non‐pregnant women. Pregnancy‐associated changes in weight or BMI did not influence terminal CAB‐LA PK. These data suggest that CAB‐LA dose adjustments are unlikely to be needed during pregnancy, but this needs to be verified in studies where CAB‐LA is administered throughout pregnancy. No published clinical data exists on infant exposure to CAB‐LA through breast milk. In pre‐clinical studies of lactating rats, CAB‐LA was detected in milk and the plasma of nursing pups. No effects were seen on lactation or on offspring growth and development [4]. Breast milk exposures are anticipated to be similar to those observed for dolutegravir (DTG); the median ratio of DTG in breast milk to maternal plasma is low (3%), and no infant AEs have been reported for breastfeeding mothers on DTG [27].

## 5 CONCLUSIONS

All these data are critical to support guidelines for the use of CAB‐LA in pregnant and lactating people, particularly in areas of high HIV prevalence where total fertility rates are high, and women wish to conceive without fear of HIV or adverse health and pregnancy outcomes. The ongoing HPTN 084 pregnancy and infant sub‐study will assess maternal and infant CAB‐LA concentrations in mother‐infant pairs where the mother continues injections throughout pregnancy and lactation. Ultimately, post‐licensure surveillance will be needed to exclude the risks of rare events like neural tube defects.

## COMPETING INTERESTS

The authors have no competing interests to declare, except SLF and ARR who are paid employees of ViiV Healthcare, and JR who is a paid employee of Gilead Sciences.

## AUTHORS’ CONTRIBUTIONS

All authors participated in the study, contributed to manuscript preparation and provided critical comments on the final manuscript. SD‐M, MAM, BH, MCH and MSC designed this analysis; CN, PM, PNN, JM, MB‐M and NM supervised protocol implementation with support from RB and JF. MAM, EP‐M, CWH and SLF provided laboratory support and interpretation; XG and BH provided data management and statistical support; JR and ARR provided pharmaceutical support; and AA provided sponsor safety oversight. RJL contributed data from HPTN 077.

## FUNDING

This study was made possible through funding support from the National Institute of Allergy and Infectious Diseases (NIAID), Office of the Director (OD), National Institutes of Health (NIH), National Institute on Drug Abuse (NIDA) and the National Institute of Mental Health (NIMH) under Award Numbers UM1AI068619 (HPTN Leadership and Operations Center), UM1AI068617 (HPTN Statistical and Data Management Center) and UM1AI068613 (HPTN Laboratory Center). Additional funding was provided by the Bill & Melinda Gates Foundation (OPP1154174) and ViiV Healthcare. Pharmaceutical support was provided by ViiV Healthcare and Gilead Sciences. The funding source played no role in the data collection or analysis of this manuscript. The authors had final responsibility for the decision to submit for publication.

## DISCLAIMER

The content is solely the responsibility of the authors and does not necessarily represent the official views of the National Institutes of Health.

## ETHICS APPROVAL STATEMENT

The HPTN 084 protocol was reviewed and approved by research ethics committees/institutional review boards and national drug authorities at all 20 participating sites prior to the implementation of activities. All participants provided written consent in a language of their choice prior to any study procedures.

## CLINICAL TRIAL REGISTRATION

NCT03164564

## Data Availability

Data collected for this study may be made available on request. Data archive will be held at Fred Hutch Cancer Center, Seattle, WA. Requests can be sent to HPTN‐Data‐Access@scharp.org.
